# One‐Dimensional Ferroelectric Nanostructures: Synthesis, Properties, and Applications

**DOI:** 10.1002/advs.201500358

**Published:** 2016-02-25

**Authors:** Longyue Liang, Xueliang Kang, Yuanhua Sang, Hong Liu

**Affiliations:** ^1^ State Key Laboratory of Crystal MaterialsShandong University27 Shandanan RoadJinan250100P.R. China

**Keywords:** application, ferroelectric, one‐dimensional, property, synthesis

## Abstract

One‐dimensional (1D) ferroelectric nanostructures, such as nanowires, nanorods, nanotubes, nanobelts, and nanofibers, have been studied with increasing intensity in recent years. Because of their excellent ferroelectric, ferroelastic, pyroelectric, piezoelectric, inverse piezoelectric, ferroelectric‐photovoltaic (FE‐PV), and other unique physical properties, 1D ferroelectric nanostructures have been widely used in energy‐harvesting devices, nonvolatile random access memory applications, nanoelectromechanical systems, advanced sensors, FE‐PV devices, and photocatalysis mechanisms. This review summarizes the current state of 1D ferroelectric nanostructures and provides an overview of the synthesis methods, properties, and practical applications of 1D nanostructures. Finally, the prospects for future investigations are outlined.

## 1 Introduction

Ferroelectric materials have been extensively studied because of their excellent ferroelectric, ferroelastic, pyroelectric, piezoelectric, inverse piezoelectric and other unique physical properties, such as mechanic‐electric‐thermal and electric‐acousto‐optic coupling properties, nonlinear optical properties, switching characteristics, FE‐PV effect, etc., which have facilitated the broad use of ferroelectric materials in various applications.^1^ Devices based on ferroelectric materials, which present high levels of sensitivity and reliability,^2^ are beneficial in various applications. However, ferroelectric materials do not easily form thin films and are generally brittle, incompatible with semiconductors or metals, and easily fractured and fatigued with mechanical and electric loads, and these properties significantly affect their potential applications.^3^ Numerous efforts have been made to resolve these issues. Previous studies have presented doping modifications, preparation process optimizations, and dimension lowering strategies as the most effective methods of improving the performance of these materials. Among this class of materials, low‐dimension ferroelectric materials have become widely studied within the field ferroelectric materials research.

Nanostructured materials less than 100 nm in one dimension, exhibit new physical phenomena because of finite size effects that occur during down‐scaling.^4^ Relative to their bulk counterparts, nanostructures cover relatively high specific surface areas, indicating the presence of a greater amount of surface atoms and higher levels of surface energy. These characteristics endow low‐dimension ferroelectric materials with specific property modifications in phase transition or Curie temperature (*T*_C_), dielectric constants, coercive fields, spontaneous polarization levels, and piezoelectric response levels.^5^ Recently, one‐dimensional (1D) ferroelectric nanostructures (wires, rods, tubes, belts, and fibers) have been extensively studied because of their specific ferroelectric behaviors related to 1D morphologies. Generally, the specific properties of 1D ferroelectric nanostructures are attributed to the increased surface area. Moreover, decrease in size and dimensionality can facilitate the formation of single domain structures, which can dramatically enhance ferroelectric properties. Therefore, 1D ferroelectric nanostructures present great potential for use in nonvolatile memory devices, microelectromechanical systems, FE‐PV devices, nonlinear optics, nanogenerators, and sensors. In recent years, considerable progress has been achieved in the study of 1D nanostructured ferroelectrics (e.g., synthesis, properties, and applications), although barriers to the practical application of nanodevices remain. Therefore, it is essential to provide a review of this promising novel nanostructure form. Although an excellent review paper on 1D perovskite ferroelectric nanostructures was published in 2011, the authors of this paper focused on ferroelectrics with perovskite structures and only addressed material synthesis issues.^6^ In recent years, although improvements to 1D perovskite‐based ferroelectric nanostructures have been achieved, several other types of 1D ferroelectric nanostructures have also proposed. Most importantly, researchers have focused on the basic properties and applications of 1D ferroelectric nanostructures, and several breakthroughs have been achieved. Thus, in this review, we provide a brief review of 1D ferroelectric nanostructures, divided into the following sections. In Section 2, we discuss the materials used in ferroelectric nanostructures, and in Section 3, we present methods of synthesizing ferroelectric nanostructures. In Section 4, we detail the properties and applications of ferroelectric materials, and in Section 5, we offer our conclusions and suggestions for future research.

## 2 Ferroelectric Materials and 1D Ferroelectric Nanostructures

### 2.1 Ferroelectric Properties and Materials

**Figure 1:**
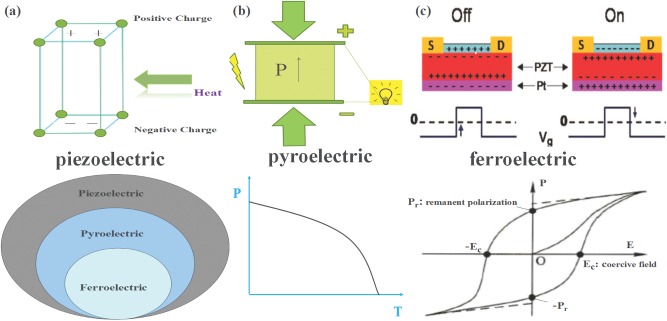
a) Venn diagram showing that all ferroelectric materials are pyroelectric and piezoelectric, but not vice versa. b,c) Graphs depicting polarization (*P*) changes that occur with respect to temperatures (*T*) and electric fields (*E*). c) Reproduced with permission.^12^ Copyright 2009, American Chemical Society.

To clarify what constitutes a ferroelectric material, and detail the specific properties of 1D ferroelectric materials, a number of concepts must be defined. Piezoelectricity^7^ is generated when single crystal materials generate electric charges of equal magnitude and with opposite symbols on the surface of a crystal when it is either subjected to directional stress or tension with the induced electrical field parallel to the stress direction, or under hydrostatic stresses. Pyroelectricity^8^ refers to electric charge generation mechanisms along specific crystallographic directions of crystals undergoing temperature changes. Ferroelectricity^9^ refers to processes of spontaneous polarization induced by asymmetric crystal structures. Asymmetric crystal structures can induce nonoverlapping charges in unit cell center of certain dielectric crystals. These charges form the electric dipole moment, which is referred to as spontaneous polarization. Spontaneous polarization capacities constitute a number of the most important factors when identifying ferroelectric material properties. The mechanisms of piezoelectricity, pyroelectricity, and ferroelectricity are illustrated in **Figure** **1**. As is well known, crystal properties are derived from crystal structures and compositions. Materials with piezoelectricity should exhibit a basic crystal structure without a center‐of‐symmetry (“noncentrosymmetric”). Moreover, pyroelectric properties require that crystal structures be noncentrosymmetric while also possessing a unique axis of symmetry (rendering them “polar” structures). Based on the crystal structures, only 20 of 32 crystal classes undergo a polarization change when subjected to mechanical stress, thus rendering them piezoelectric. Ten of these 20 classes possess a unique axis of symmetry (polar) and electric polarization within their structures in the absence of an applied field, which represents the fundamental structure of pyroelectric properties. Ferroelectrics are a subgroup of pyroelectrics, wherein spontaneous polarization within the structure can alternates between different stable directions through the application of a sufficient magnitude electric field (coercive field).^10^ Depending on their compositions, ferroelectric materials can be assigned to three categories: (1) inorganic oxides; (2) inorganic nonoxides; and (3) organic crystals, liquid crystals, and polymers. The ABO_3_ perovskite‐type family, such as barium titanate (BaTiO_3_), lead titanate (PbTiO_3_), alkaline niobate (KNbO_3_, LiNbO_3_), and zirconate titanate (PbZr*_x_*Ti_(1−_ *_x_*_)_O_3_, which is abbreviated to PZT), are the oxide‐based ferroelectrics that have attracted the most attention.^4^ A more detailed description of different ferroelectric families can be found elsewhere.^11^

The ferroelectricity properties of single‐crystal materials (Rochelle salt) were discovered in 1921^13^ and subsequently extended to the field of polycrystalline ceramics (BaTiO_3_) during the early to mid‐1940s,^14^ a number of industrial and commercial applications of bulk ferroelectric materials have been realized, such as high dielectric‐constant capacitors, piezoelectric sonar and ultrasonic transducers, radio and communication filters, pyroelectric security surveillance devices, infrared imaging, medical diagnostic transducers, positive temperature coefficient sensors and switches devices, ultrasonic motors, electro‐optic light valves, thin‐film capacitors, and ferroelectric thin‐film memories.^15^ Besides, periodically poled structures in ferroelectrics, especially in LiNbO_3_ single crystals, have attracted considerable attention because of their potential applications in nonlinear optical processes, such as second harmonic generation (SHG), sum/difference frequency, and optical parametric oscillation (OPO).^16^ Recently, approaches to bulk functional material nanocrystallization have provided opportunities to generate novel materials and devices, and ferroelectric nanostructures, have attracted the attention of materials scientists, materials chemists, applied physicists, and electronic engineers.

### 2.2 Ferroelectric Nanostructures and 1D Ferroelectric Nanostructures

Because of the nanoeffects of the nanostructures, including the quantum size effect, quantum confinement effect, and surface effect, ferroelectric nanostructures have attracted considerable attention for a number of years.^17^ Low‐dimensional ferroelectric nanostructures are composed of 0‐dimensional granular nanoparticles (NPs), 1D nanowires (NWs), nanotubes (NTs), and 2D nanofilms (NFs).^18^ Because ferroelectric NFs present low voltage conversion levels, higher optical‐electric sensibility levels, and stronger optical confinement properties relative to that of bulk ferroelectric materials, they may prove useful when applied in ferroelectric random access memory (FeRAM), dynamic random access memory (DRAM), infrared imaging, and optical waveguide devices.^19^ 0D ferroelectric NPs present certain unique properties, such as stronger piezoelectric properties and an intrinsic single domain. However, assembly difficulty complications constitute obstacles to their practical application.^20^ As predicted, when the radial size of 1D nanostructures is smaller than 100 nm, considerably lower spontaneous polarization conversion voltage levels along the radial direction enhance the polarization of 1D ferroelectric nanostructures. Polarized 1D ferroelectric materials can be used in data storage and optical applications (e.g., SHG and OPO^17^, ^21^). Because of their considerably higher piezoelectric efficiency and mechanical strain tolerance levels, 1D ferroelectric nanostructures, particularly vertically or horizontally aligned arrays of piezoelectric NWs, serve as ideal configurations for energy harvesting applications.^22^

## 3 Synthesis of 1D Ferroelectric Nanostructures

A number of synthesis approaches have been developed and applied to create 1D ferroelectric nanostructures with different compositions, structures, and target applications. In this section, the typical synthesis approaches are described to provide a brief overview of the material preparation methods.

### 3.1 Hydrothermal Methods

The hydrothermal method is one of the most popular approaches to synthesize nanomaterial. This method typically involves a heterogeneous chemical reaction in aqueous media above room temperature (normally exceeding 100 °C) under pressure levels higher than 1 atm^23^ and the increased reactant solubility under these high pressures generates unusual reactions.^24^

Typical hydrothermal processes involve precursor synthesis and reactions in an autoclave oven at a certain temperature. In certain cases, a product must undergo post‐treatment to complete a crystal structure transition.^25^ Morphological control requires various precursors, heat‐treatments, and surfactants during synthesis.^26^ This approach facilitates the easy diffusion and homogeneous nucleation of the reacting species while also allowing for the formation of highly crystalline stoichiometric materials at low temperatures.

**Figure 2:**
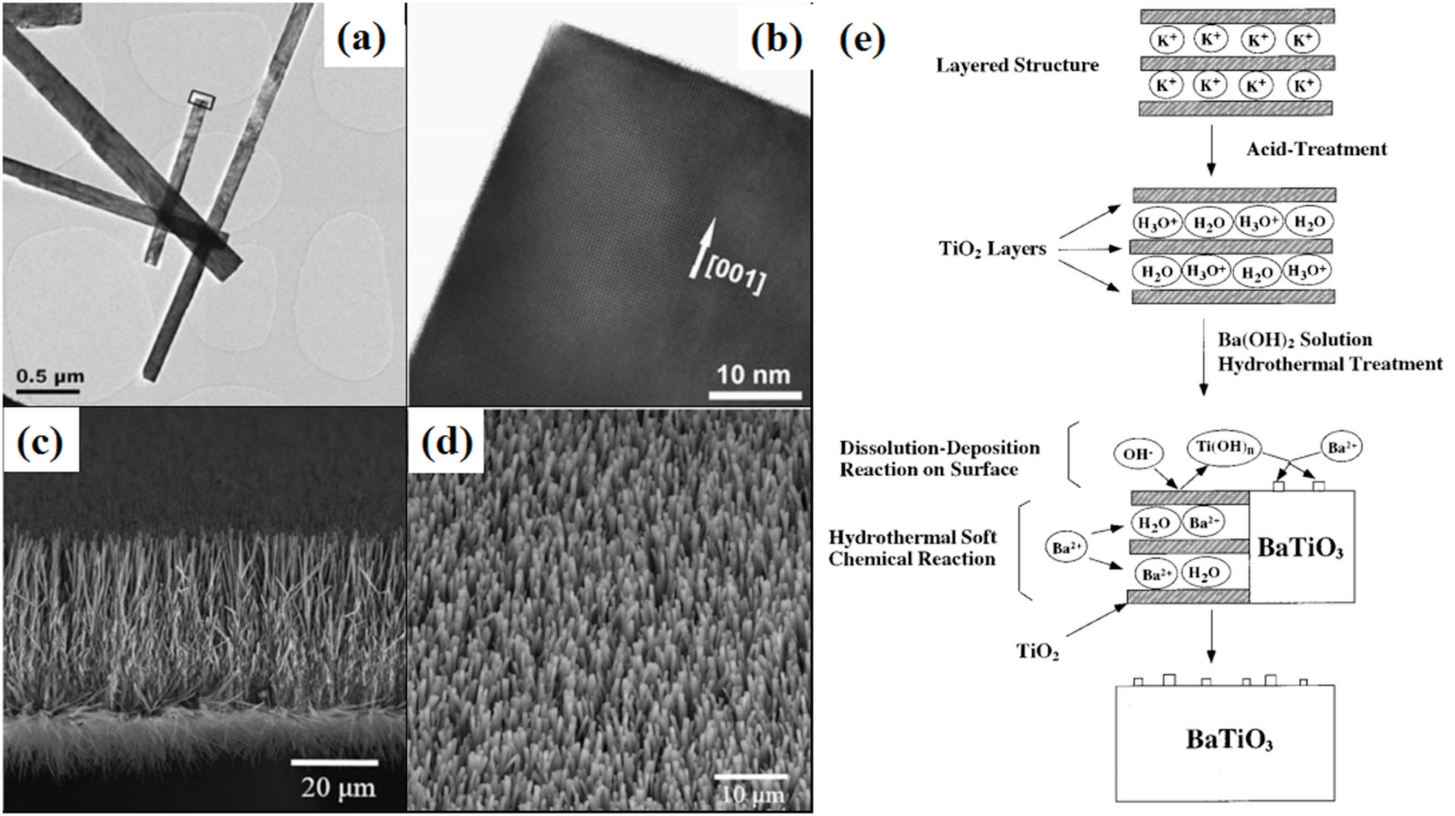
a) TEM (transmission electron microscopy) image of KNbO_3_ NRs synthesized at 180 °C for 48 h using SDS (0.005 mol). b) HRTEM (high‐resolution transmission electron microscopy) image of the tip of the rod pictured in (a); c) Textured TiO_2_ film used as the nucleation sight for highly aligned NWs (cross‐sectional view). d) SEM (scanning electron microscope) images of the BaTiO_3_ nanofibers; e) A model of the formation reactions of BaTiO_3_ using layered titanate. a,b) Reproduced with permission.^28^ Copyright 2009, Royal Society of Chemistry. c,d) Reproduced with permission.^29^ Copyright 2015, American Institute of Physics. Reproduced with permission.^30^ Copyright 2001, American Chemical Society.

Using the hydrothermal method, titanate and niobate 1D nanomaterials have been obtained.^27^ However, the hydrothermal processes required to create well‐crystalline KNbO_3_ NRs and NWs can take as long as a week. Recently, Wang et al.^28^ synthesized single‐crystalline KNbO_3_ NRs via Nb_2_O_5_ hydrothermal treatments at 180 °C for 24 h using a KOH solution with a sodium dodecyl sulfate (SDS) surfactant. The morphology of the KNbO_3_ product was strongly affected by the addition of the surfactant. Under optimized reaction conditions (e.g., reactant concentrations, hydrothermal temperature, and reaction time), orthorhombic KNbO_3_ NRs (100–300 nm in diameter, and up to 5 mm in length), growing along the [001] direction were successfully synthesized (**Figure** **2**]a,b).

In addition, Kim et al.^31^ used a hydrothermal method to synthesize KNbO_3_ NWs with a monoclinic crystal phase. The thicknesses and lengths of the NWs were 74.0 ± 11.7 nm and 5.1 ± 1.4 μm, respectively. When using metallic Nb and replacing Nb_2_O_5_ powder as the reactant, higher levels of active metallic Nb than those in Nb_2_O_5_ were attributed to a only 12 h reaction time. Similarly, when Nb_2_O_5_ and LiOH powder were used as the Nb and Li sources, respectively, LiNbO_3_ NWs were obtained at a low temperature.[[qv: 27d]]

BaTiO_3_ and SrTiO_3_ are important members of the ABO_3_ perovskite‐type ferroelectric family; however, it is difficult to obtain BaTiO_3_/SrTiO_3_ 1D nanostructures because of their highly symmetric cubic structures at synthesis temperatures. Mao et al.^32^ successfully synthesized BaTiO_3_/SrTiO_3_ NTs using a simple low‐temperature hydrothermal method that involved the use of titanium oxide (TiO_2_) NTs as a precursor and template. Reactants with an initial 1:1 molar ratio of TiO_2_ NTs to Ba(OH)_2_ or SrCl_2_ were subsequently refluxed in an oil bath for 20–60 h with stirring under argon or nitrogen conditions. The outer diameters of the BaTiO_3_ and SrTiO_3_ NTs were in the range of 8 to 15 nm, the inner diameters ranged from 4 to 7 nm, and the length varied from 50 to over 500 nm. Bowland et al.^29^ recently developed a synthesis methodology for producing high aspect ratio and highly aligned ferroelectric BaTiO_3_ NW arrays via versatile hydrothermal reactions. A textured TiO_2_ film, was synthesized through a two‐step hydrothermal reaction involve the use of titanium foil as a reactant, and it allowed the competitive growth process to initiate nucleation in a preferred orientation. Sodium titanate was hydrothermally grown on the TiO_2_ film, and then completely converted into BaTiO_3_ via an ion diffusion mechanism (Figure 2c,d). Similarly, Tian et al.^33^ used sodium titanate nanofibers as reactants obtained via hydrothermal methods, and they reported the synthesis of BaTiO_3_/SrTiO_3_ nanofibers based of an ion‐exchange process. In brief, TiO_2_ powder was reacted with NaOH in a sealed autoclave maintained at 240 °C for 3 d to form sodium titanate nanofibers. Because of its layered structure, the interlayered Na^+^ between the [TiO_6_] octahedral layers can be replaced by Sr (II) or Ba (II) through a similar hydrothermal process. Figure 2e shows a model of the formation reactions of BaTiO_3_ using layered titanate. Moreover, the hydrothermal method can be applied to develop many other types of 1D multiferroelectric nanostructures, such as BiFeO_3_ NWs and PbHPO_4_ NWs.^34^

The hydrothermal method is an easy and low‐cost method of synthesizing of 1D ferroelectric nanostructures. In addition to the effect of intrinsic nanocrystal growth under certain conditions, the raw reactant properties, surfactant additions, and template may have a significant influence on the morphology of nanomaterials. Therefore, numerous 1D ferroelectric nanostructures can be synthesized using the hydrothermal method. One drawback of using this method is the small quantities of product that can be synthesized. To address this problem, efforts have been made to scale‐up hydrothermal reactions toward the mass‐production of 1D ferroelectric nanostructures.^35^

### 3.2 Sol–Gel Template Methods

**Figure 3:**
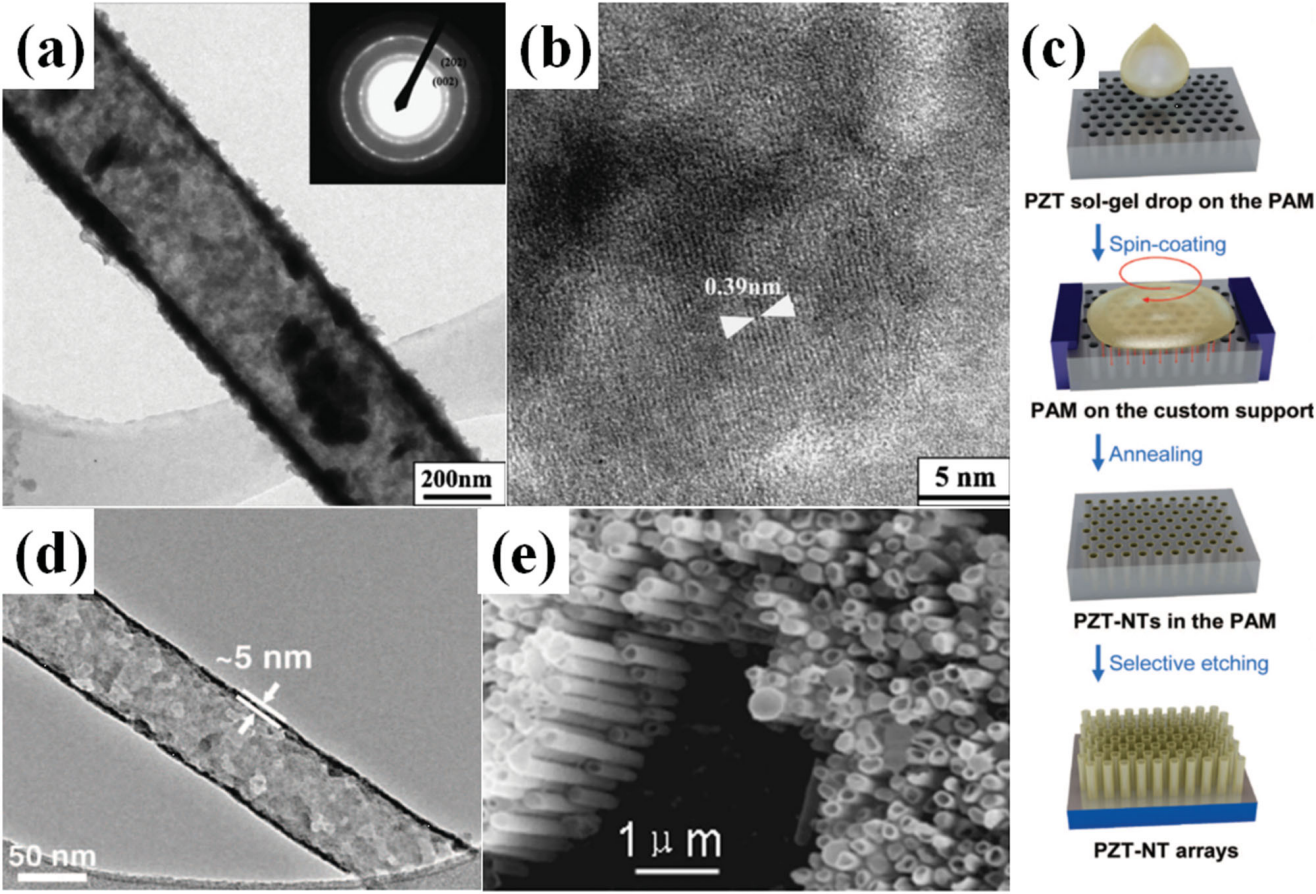
a) TEM image of the prepared PTO NTs. Inset: SAED (selected‐area electron diffraction) pattern of the PTO NTs. b) HRTEM image of the crystallized region of the prepared PTO NTs; c) Schematic illustration of the PZT NT array synthesis procedure. d) Individual PZT NT with a wall thickness of ≈5 nm; e) SEM images of the BFO NTs. a,b) Reproduced with permission.^36^ Copyright 2008, Elsevier. c,d) Reproduced with permission.^39^ Copyright 2008, American Chemistry Society. e) Reproduced with permission.^44^ Copyright 2005, American Institute of Physics.

Sol–gel template synthesis has been identified as a simple, low‐cost method of fabricating complex oxide 1D ferroelectric NWs and NTs. In 2008, PbTiO_3_ NT arrays,^36^ and PZT NWs^37^ were synthesized via sol–gel template methods that involved filling PbTiO_3_ or PZT precursor sol in the channel of anodic aluminum oxide (AAO). Subsequent to calcination for crystallization, the template was removed through immersion in 4 m NaOH solution for 6 h.^37^ The diameters and lengths of the PbTiO_3_ NTs were roughly 300 nm and 50 mm, respectively, and most presented a wall thickness of several tens of nanometers (**Figure** **3**a,b). Moreover, strain effects on the temperature‐dependent photoluminescence properties in the clamped (with template) and free‐standing (without template) PbTiO_3_ NTs were examined.^38^ Using a nanochannel AAO as a template, 45 nm diameter PZT NWs in diameter were synthesized. Thus, the diameter of AAO determines the diameter of the synthesized NTs and NWs.

In 2008, Kim et al.^39^ fabricated PZT NTs via a template‐directed method that involved integrating sol–gel processes via spin‐coating techniques, and porous alumina membrane (PAM) templates. The wall thickness and outer diameter of each PZT NT were ≈5 and 50 nm, respectively (Figure 3c,d). In 2013, a similar template‐directed method was used to synthesize 1D lead‐free ferroelectric nanostructures and BaTiO_3_‐substituted BiTiO_3_ ((Bi_0.5_Na_0.5_)_0.92_Ba_0.08_TiO_3_, BNT−BT_0.08_) NTs, and a porous polycarbonate membrane was used as a template.^40^ The average BNT−BT_0.08_ NTs were ≈650 and 50 nm in outer diameter and wall thickness, respectively. Their lengths did not vary significantly from those of the template (20 mm).

1D nanostructures of ferroelectric polymers, have high levels of mechanical flexibility and low levels of acoustic impedance, can be synthesized via a template‐assisted method with the use of anodic porous alumina (APA) membrane templates, which were obtained from the anodization of thin aluminum films or sheets.^40^ The pores are highly uniform in size and spacing (mainly arranged with a hexagonal symmetry) and open at both ends. Of the polymers studied thus far, poly(vinylidene fluoride) (PVDF) and its copolymer, poly(vinylidene difluoride–trifluoroethylene) (PVDF‐TrFE) are the most widely used in engineering applications.^42^ Depending on the process used for the APA impregnation, that is, vacuum impregnation, spin coating, or solution casting, all followed by proper thermal treatments, vertically oriented NTs or NWs showing uniform diameter distribution and very high aspect ratios have easily been obtained.^43^

In addition, Bibased complex oxide 1D perovskite nanostructures can be synthesized using this sol–gel template method with the proper raw materials. Zhang et al.^44^ successfully prepared BiFeO_3_ (BFO) NTs with diameters of ≈250 nm and lengths of ≈6 μm with a sol–gel method using nanochannel alumina templates (Figure 3e). More complex 1D ferroelectric nanostructures, high‐ordered rare earth‐doped BFO, and Bi_0.85_Nd_0.15_FeO_3_ (BNF) NT arrays were also synthesized by Guo et al. using a similar sol–gel template method.^45^ The NT arrays were 200 nm in diameter, 60 μm in length, and 20 nm in wall thickness.

The sol–gel template synthesis method is a universal method employed for the synthesis of 1D ferroelectric nanostructures, and it can also be used to synthesize most inorganic ferroelectric materials as long as an appropriate sol–gel precursor is included. The morphology of the 1D ferroelectric nanostructures is inherited from the channel of AAO templates; therefore, the diameters and lengths of the NTs or NWs are uniform. Unfortunately, the NTs or NWs synthesized with this method are normally polycrystalline and possess physical properties that are difficult to develop. When the physical function of the 1D ferroelectric nanostructures is essential, this method should not be used or the current sol–gel template synthesis methods should be improved to support the generation of single crystalline materials.

### 3.3 Nanosolid‐State Reaction Methods

The solid‐state reaction method is an important method of synthesizing inorganic powders or sintering inorganic ceramics. During a solid‐state reaction process, two or more compounds or elementary substances are mixed, and react in the solid state at high temperatures, thereby generating new materials with bulk or powder morphologies. Solid‐state reactions have been widely used in several industries, such as high temperature ceramics, electronic ceramics, and even super‐conducting materials.^46^

The nanosolid‐state reaction method is a modified solid‐state reaction method that is used for synthesizing nanostructures. Compared with conventional solid‐state methods, the nanosolid‐state reaction method utilizes a nanosized system. Normally, crystalline nanostructures, (e.g., NW, NB (nanobelt), and NP), include a second phase layer covering the nanostructure surface to form a core–shell nanostructure. By applying a heat‐treatment, a new crystalline phase forms from the solid‐state reaction between the core and shell, which inherits the morphology of the core. During the synthesis process, the core acts as a raw material as well as a template. This method can synthesize nanostructures with crystallization habits that are not suitable for use in morphologically controllable synthesis.^47^

Layered perovskite‐like bismuth titanate (Bi_4_Ti_3_O_12_, which is abbreviated to BIT) and its derivatives are important ferroelectric materials with high Curie temperatures and low ferroelectric fatigue levels, thus allowing for their broad application in numerous areas (e.g., capacitors, sensors, and nonvolatile memory devices).^48^ However, 1D bismuth titanate nanostructures are difficult to synthesize using conventional wet‐chemical and physical deposition methods. Buscaglia et al.^49^ developed two types of nanosolid‐state reaction methods for synthesizing 1D Bi_4_Ti_3_O_12_ nanostructures. One method involves the use of BiO*_x_* NRs as the template and core, and H_2_Ti_3_O_7_ as the shell. During nanosolid‐state reaction, the Bi precursor migrates outward and reacts with H_2_Ti_3_O_7_ to form hollow 1D Bi_4_Ti_3_O_12_ NTs. The other method utilizes the reverse process, with H_2_Ti_3_O_7_ as template and core, and Bi_2_CO_5_ as the shell. Subsequent to the nanosolid‐state reactions between H_2_Ti_3_O_7_ and Bi_2_CO_5_, Bi_4_Ti_3_O_12_ NRs were obtained.

**Figure 4:**
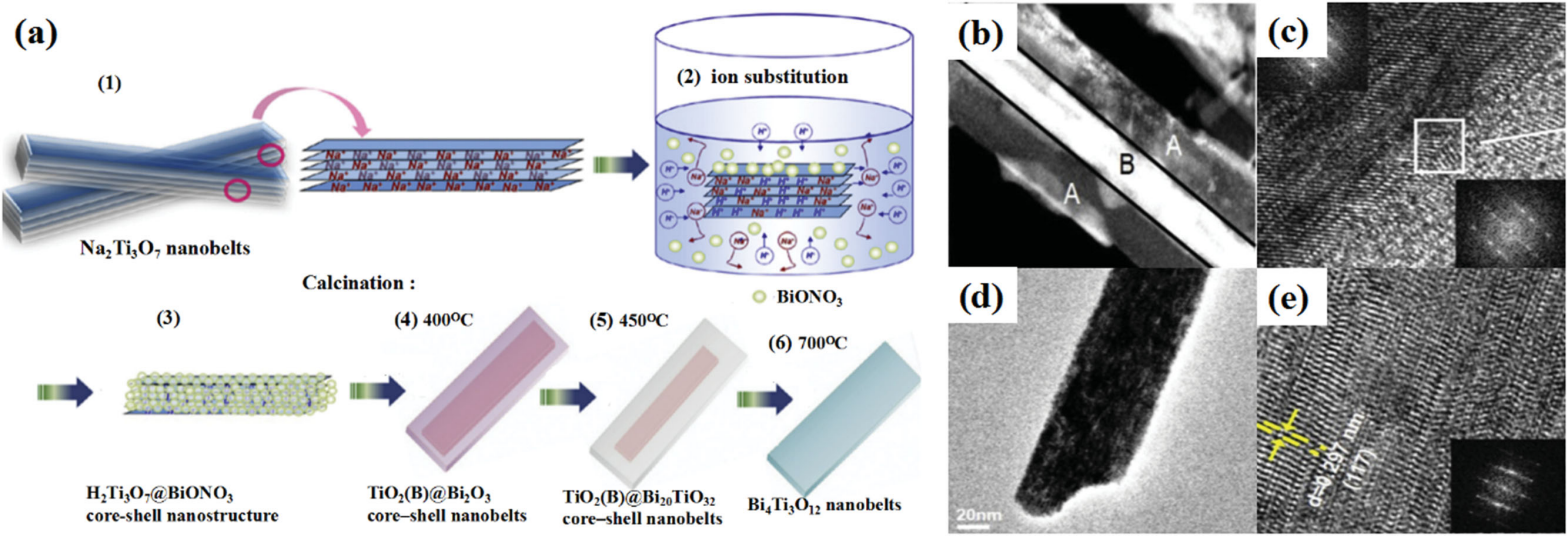
a) Schematic of the reaction and phase transformation mechanism of bismuth titanate NBs. Representative (HR)TEM images of b,c) TiO_2_(B)@Bi_20_TiO_32_ NBs that were prepared by calcination at 450 °C and d,e) Bi_4_Ti_3_O_12_ NBs that were prepared by calcination at 700 °C. Reproduced with permission.^50^ Copyright 2014, Elsevier.

Hao et al.^50^ developed an effective low‐temperature method for the synthesis of Bi_4_Ti_3_O_12_ NBs by using Na_2_Ti_3_O_7_ NBs as the reactants and templates. The experimental procedure including an ion substitution followed by a nanoscale solid‐state reaction. First, Na_2_Ti_3_O_7_ NBs were soaked in a bismuth nitrate solution where ion substitution at the NB surfaces forming the H_2_Ti_3_O_7_@BiONO_3_ core–shell structure, which were then postcalcined at various temperatures. At the calcination temperature of 400 °C, the top layer was converted to Bi_2_O_3_ whereas the interior was converted to TiO_2_(B), forming TiO_2_(B)@Bi_2_O_3_ core–shell NBs. When the calcination temperature increased to 450 °C, a metastable interphase Bi_20_TiO_32_ formed on the NB surface whereas the interior structure remained virtually unchanged, and the NBs now exhibited the TiO_2_(B)@Bi_20_TiO_32_ core–shell structure. At calcination temperatures higher than 550 °C, the shell of the NBs became Bi_4_Ti_3_O_12_. At even higher temperatures (600–700 °C), no TiO_2_(B) was detected and the NBs exhibited single‐crystalline characteristics that were consistent with those of Bi_4_Ti_3_O_12_ (**Figure** **4**).

Besides, single crystal BaTiO_3_ NWs with widths between 50 and 300 nm and lengths between 2 and 30 μm can be obtained through an original solid‐state process using layered TiO_2_ NWs coated with BaCO_3_ nanocrystals as the reactive templates.^51^

The nanosolid‐state reaction method is a universal method for synthesizing several 1D complex oxide‐based ferroelectric nanostructures with appropriate reactants of specific morphologies. However, similar to the sol–gel template method, this method rarely synthesizes single crystalline 1D nanostructures. Although this type of 1D ferroelectric nanostructures can be used as a photocatalyst, it is not suitable for use in ferroelectric nanodevices.

### 3.4 Molten Salt Methods

The molten salt method is derived from the flux method for bulk single crystals growth. In this method, two or more reactants are dissolved in high‐temperature molten salt, and the reactants then form dissoluble complex materials. By altering the solute concentrations and reaction temperatures, the nucleation and growth processes of complex materials can be controlled and 1D nanostructures can be obtained.^36^ Alkaline hydroxides, possess a low melting point and are widely used as flux for the synthesis of complex oxide nanostructures. Specifically, mixtures of NaOH and KOH (Na/K ratio of 51.5:48.5) with a low melting point of 150 °C have been specially studied via the composite‐hydroxide mediate synthesis method.^52^

Potassium niobate with K_2_Nb_8_O_21_ was first studied by Guerchais in 1962^53^ and is well known for its strong dielectric properties and high levels of photocatalytic activity.^54^ Recently, Xu et al.^55^ successfully prepared single crystalline K_2_Nb_8_O_21_ nanoribbons using a simple molten salt method without employing a particular atmosphere or any surfactant. In brief, a mixture of Nb_2_O_5_ and KCl powder was ground for 10 min and then calcined in a tube furnace at 800 °C for 3 h. The chemical reaction for synthesizing K_2_Nb_8_O_21_ is described by the following formula^55^

(1) 8Nb2O5(s)+4 KCl (s)+O2(g)→2K2Nb8O21(s)+2Cl2(g)

**Figure 5:**
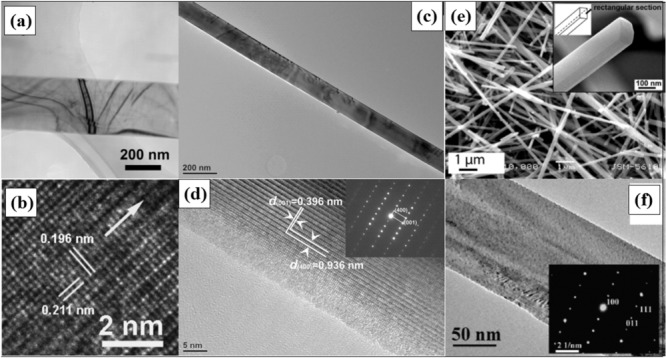
a) TEM and b) HRTEM images of an individual K_2_Nb_8_O_21_ nanoribbon; c) TEM and d) HRTEM images of the a K_2_Nb_8_O_21_ NWs; e) SEM image of the synthesized BaTi_2_O_5_ NWs (870 °C, 9 h). f) HRTEM of a single BaTi_2_O_5_ NW; Inset is the corresponding SAED pattern. a,b) Reproduced with permission.^55^ Copyright 2008, Elsevier. c,d) Reproduced with permission.^56^ e,f) Reproduced with permission.^58^ Copyright 2010, American Chemical Society.

The K_2_Nb_8_O_21_ nanoribbons are 100—500 nm in width, 30 nm in thickness, and tens of micrometer in length (**Figure** **5**a,b). With increases in the calcination temperatures, K_2_Nb_8_O_21_ NWs forms instead of nanoribbons.^56^ After calcination at 900 °C or 1000 °C for 3 h, the typical widths and lengths of the resulting K_2_Nb_8_O_21_ NWs are ≈130 nm and 20 μm, respectively (Figure 5c,d). Moreover, Xu et al.^57^ prepared single‐crystal NaNbO_3_ and CaNb_2_O_6_ NRs through molten‐salt reaction using K_2_Nb_8_O_21_ NWs as a template in molten sodium and calcium salts, respectively.

Other important 1D ferroelectric materials, that are difficult to obtain through other methods, have been obtained through the molten salt method. For instance, Deng et al.^58^ synthesized single‐crystalline BaTi_2_O_5_ NWs with BaC_2_O_4_•H_2_O and TiO_2_ powders used as the precursors (Figure 5e,f). The BaMnO_3_ NRs were synthesized using mixed NaOH and KOH with a Na/K ratio of 51.5:48.5 as the flux, and BaCl_2_, and MnO_2_ as the raw materials. The synthesis temperature was as low as only 180 °C.^59^

The molten salt method constitutes a powerful approach to synthesizing 1D ferroelectric nanostructures. This one‐step synthesis process provides for easy and reliable synthesis, and composite‐hydroxide mediated synthesis methods can be performed at low temperatures, thus rendering synthesis cost effective and convenient. The single crystalline characteristics of the synthesized 1D nanostructures endow the material with perfect physical properties, thus improving device applications. However, this method is to a large extent limited to the growth of 1D materials with anisotropic structure. Interactions and absorption of specific ions from the molten salt on specific crystallographic faces can tune the growth direction by blocking growth incertain directions, thus regulate the nanostructure morphologies.^35^ Therefore, additional efforts should be made to study the effects of flux on the crystallization process. With further refinement, this method has potential for use as a universal method of synthesizing several materials with specific morphologies through flux modifications.

### 3.5 Electrospinning Methods

Electrospinning has been exploited to produce fibers with polymeric, composite, and ceramic compositions. In this method, high voltage is applied to a precursor solution or melt as it is ejected from a metallic needle. The droplet that forms at the tip of the needle is deformed by electrostatic forces into a conical shape known as a Taylor cone. When the solution properties are properly tuned, repulsion between the surface charges produces a stable jet that undergoes whipping and deformation and produces thin fibers with nanoscale diameters.^60^ The electrospinning method can be used to prepare nano/microfibers with different compositions. Organic nanofibers can be directly obtained from electrospinning processes, whereas inorganic nanofibers must undergo a postcalcination process after electrospinning process.

**Figure 6:**
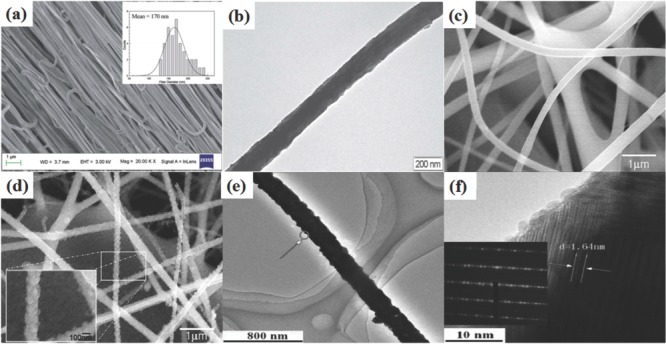
Micrographs of fibers obtained from the 18 wt% PVDF solution: a) SEM and b) TEM images. The inset shows the fiber size distribution. c) SEM image of the synthesized BaTiO_3_ and PVP composite nanofibers. d) SEM image of BaTiO_3_ nanofibers annealed at 750 °C for 1 h. The inset shows the polycrystalline fiber morphology; e) Representative TEM image of the single BNT nanofiber. f) HRTEM image of the selected area marked by an arrow in the TEM image. The inset shows the corresponding SAED pattern. a,b) Reproduced with permission.^63^ Copyright 2011, Royal Society of Chemistry. c,d) Reproduced with permission.[[qv: 68a]] Copyright 2005, Elsevier. e,f) Reproduced with permission.[[qv: 72a]] Copyright 2010, American Institute of Physics.

PVDF and PVDF–TrFE nanofibers are conveniently synthesized via electrospinning. PVDF can typically crystallize in five different forms, called the α, β, γ, δ, and ε phases,^61^ among which the β‐crystalline form exhibits the highest degree of polarization, rendering it the most polar phase. The strong mechanical stretching and electrical poling during the electrospinning process can promote β‐phase formation, with the c‐axes of β‐crystallites preferably aligned along the fiber axis.^62^ Baji et al.^63^ generated PVDF nanofibers with diameters ranging from 70 to 400 nm via electrospinning and dimethyl formamide (DMF) and acetone were employed as the PVDF solvents. PVDF nanofibers of 70 ± 17, 170 ± 31, and 400 ± 40 nm in diameter can be obtained by controlling the PVDF concentration in electrospinning solutions. Nanofibers with finer diameters include a greater amount of the β‐crystalline phase (ferroelectric phase) relative to fibers with a larger diameter. **Figure** **6**a,b shows the typical morphology of PVDF nanofibers obtained from a solution of 18 wt% PVDF. The authors then modified the route by applying extensional forces to the precursor droplet in the system. The obtained nanofibers had dipoles that were naturally aligned within the crystals. These dipoles can be aligned and reversed by applying an external electric field. The co‐polymer PVDF‐TrFE crystallizes predominantly in the β phase, readily showing an intrinsic polarization. PVDF‐TrFE is an electrically insulating ferroelectric copolymer at RT for TrFE content in the range 20%–50%.^64^ The permanent dipole moments of this copolymer are perpendicular to the main chain polymer backbone, thus affecting its ferroelectric characteristics. Mandal et al.^65^ obtained PVDF‐TrFE nanofibers with an average diameter ranging from 60 to 120 nm via electrospinning, then they elucidated the spontaneous dipole orientation during the electrospinning process by infrared spectroscopy and found that the preferential fiber orientation was along the rotation direction of the rotating substrate. 1D ferroelectric composite nanofibers^66^ and closely aligned PVDF‐TrFE nanofiber webs^67^ with high levels of uniformity and smooth surfaces were fabricated.

In 2005, Yuh et al.^68^ demonstrated an application of electrospinning method for developing 1D inorganic ferroelectric nanostructures with BaTiO_3_ nanofibers. Perovskite BaTiO_3_ nanofibers were achieved after heat treatment at 750 °C for 1 h. The diameter of a typical nanofiber ranges between 80 and 190 nm, and the length exceeds 100 μm. This study was the first describing stand‐alone electrospun complex oxide ferroelectric nanofibers (Figure 6c,d). Adjusting alkoxide precursor concentration can control the morphology of 1D BaTiO_3_ nanofibers and produce thin BaTiO_3_ fibrils or nanoribbons of <50 nm in diameter.^69^ Recently, the following 1D inorganic ferroelectric nanostructures have been prepared via the electrospinning method, barium strontium titanate (Ba_0.6_Sr_0.4_TiO_3_ or BST)^70^ nanofibers using a precursor solution of polyvinylpyrrolidone (PVP) containing BST, BiFeO_3_ nanofibers,^71^ and more complex oxide 1D ferroelectric nanostructures, such as Nd‐substituted bismuth titanate (Bi_3.15_Nd_0.85_Ti_3_O_12_, BNT) nanofibers^72^ (Figure 6e,f) and bismuth titanates with La‐substituted (Bi_3_ *_._*_25_La_0_ *_._*_75_Ti_3_O_12_, BLT) nanofibers.^73^

Thus, the electrospinning method is an efficient mass‐production method for synthesizing 1D ferroelectric nanostructures. However, the produced nanofibers are polycrystalline, which hinders their application in ferroelectric devices.

### 3.6 Specific Nonoxide Material Routes

To date, most of the studies on 1D ferroelectric nanostructures have primarily focused on oxide‐based perovskite materials and neglected nonoxide‐based materials. Because of their chemical and physical properties, most of the above methods are not suitable for synthesizing 1D nonoxide ferroelectric nanostructures; however, other synthesis methods, including the vapor phase deposition and sonochemistry methods, can be used to synthesize 1D nonoxide ferroelectric nanostructures.

Among these nonoxide based ferroelectrics, SbSI has attracted considerable attention because of the highly anisotropic behaviors of several of its functional properties, such as its high peak pyroelectric coefficient, dielectric constant, refractive index, and piezoelectric coefficient levels along the polar axis in a single crystalline form.^74^ These versatile functional properties of SbSI support its use in many applications, including thermal imaging,^75^ nonlinear optics,^76^ and mechanical actuation.^77^ However, difficulties associated with obtaining vertically aligned pure‐phase SbSI restrict its widespread application. Varghese et al.^78^ recently reported on the catalyst‐free synthesis of c‐axis oriented SbSI NR arrays on AAO substrates by vapor phase deposition. The roughness of AAO substrates plays a decisive role in the orientation control of SbSI NRs. The authors showed that the optimum temperature for obtaining pure‐phase SbSI is 250 °C and deposition temperatures lower and higher than 250 °C were found to produce SbI_3_‐rich SbSI and nonstoichiometric SbSI, respectively.

**Figure 7:**
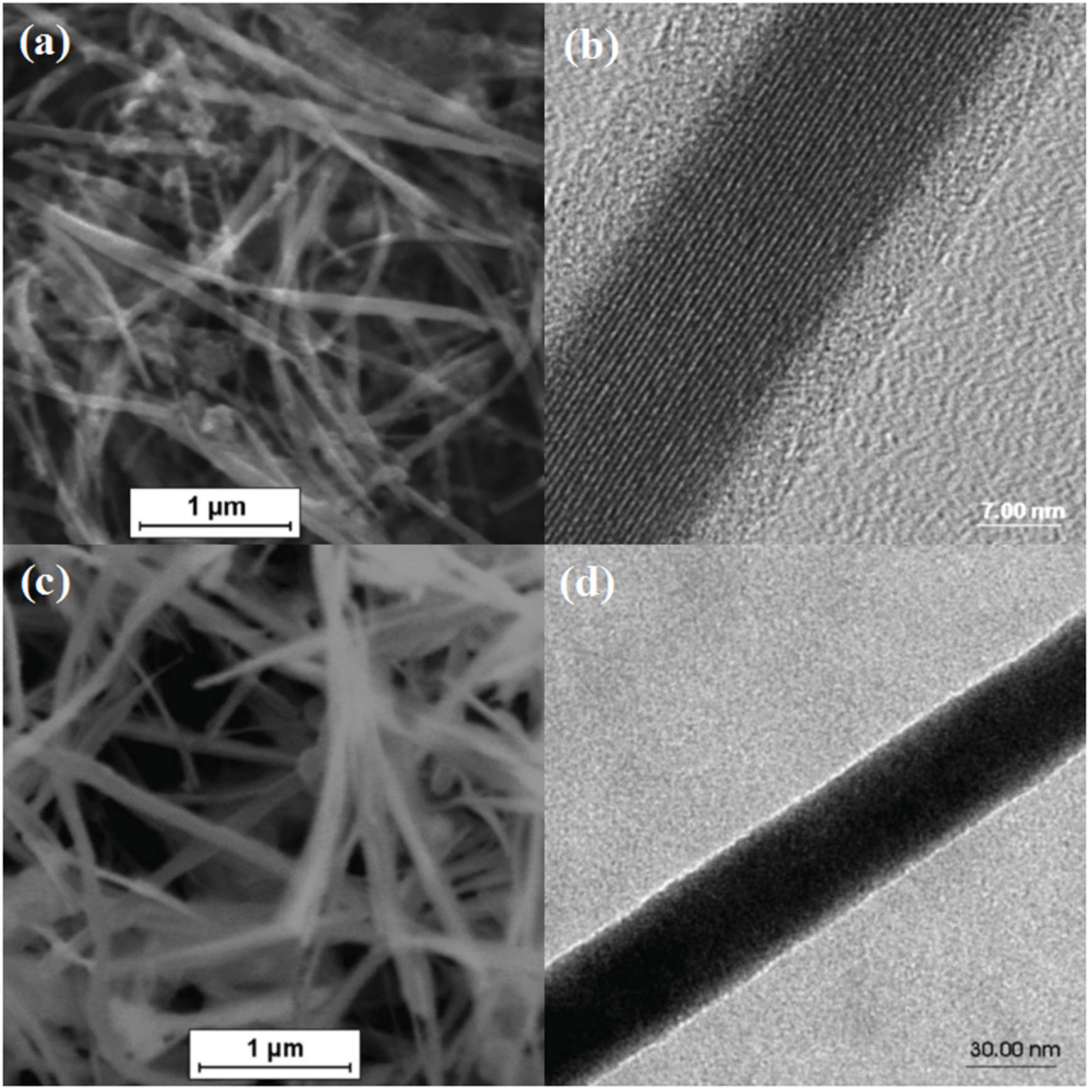
a) Typical SEM micrograph of sonochemically prepared SbSI ethanogel and b) typical HRTEM image of an individual NR from sonochemically prepared SbSI ethanogel. The fringe spacings of 0.642(2) nm correspond with interplanar distances between the (110) planes of SbSI crystal. c) Typical SEM image of dried multiwalled CNTs filled with SbSI ultrasonically submerged in methanol and d) typical TEM image of a relatively thin individual multiwalled CNT filled with SbSI ultrasonically submerged in methanol. a,b) Reproduced with permission.^79^ Copyright 2008, Elsevier. c,d) Reproduced with permission.^80^ Copyright 2009, Elsevier.

SbSI NWs were also successfully prepared via sonochemical methods,^79^ and the resulting products occurred as a porous xerogel composed of NWs with average lateral dimensions of 10–50 nm and average lengths up to several micrometers (**Figure** **7**a,b). The use of chemistry‐based ultrasound techniques can shorten reaction times and increase productivity levels.

In addition, Nowak et al.^80^ showed for the first time that SbSI can be grown in multiwalled carbon nanotubes (CNTs). SbSIs were prepared sonochemically using elemental Sb, S, and I in the presence of methanol under ultrasonic irradiation. The resulting SbSI/CNT composite exhibited a highly anisotropic 1D structure with electronic and optical properties that were heavily modified with respect to the encapsulating NT (Figure 7c,d).

To provide a brief idea overview of the synthesis methods and the materials, we have summarized the methods used to develop various 1D ferroelectric nanostructures (**Table** **1**).

**Table 1:** Summary of the synthesis methods and materials used to synthesize 1D ferroelectric nanostructures

Synthesis method	Materials
Hydrothermal methods	KNbO_3_,[[qv: 27b,28,31,81]] K_1−_ *_x_*Na*_x_*NbO_3_,^82^ LiNbO_3_,[[qv: 27d,83]] BaTiO_3_,^29^, ^31^, ^32^, ^84^ SrTiO_3_,^32^, ^33^, ^85^ Ba_1−_ *_x_*Sr*_x_*TiO_3_ 86 PbTiO_3_,^87^ PZT,^88^ Bi_3.15_Nd_0.85_Ti_3_O_12_,^89^ BiFeO_3_,[[qv: 34a]] Bi_0.9_La_0.1_FeO_3_,^90^ PbHPO_4_.[[qv: 34b]]
Sol–gel template methods	PbTiO_3_,^36^, ^38^, ^91^ BaTiO_3_,^92^ SrTiO_3_,^90^, ^93^ Ba_1−_ *_x_*Sr*_x_*TiO_3_,^94^ PZT,^37^, ^39^, ^95^ BiFeO_3_,^44^, ^96^ (Bi_0.5_Na_0.5_)_0.92_Ba_0.08_TiO_3_,^40^ Bi_0.85_Nd_0.15_FeO_3_.^45^
Nanosolid state reaction methods	Bi_4_Ti_3_O_12_,^49^, ^50^ BaTiO_3_.^51^, ^97^
Molten salt methods	K_2_Nb_8_O_21_,^55^, ^56^ NaNbO_3_,^56^, ^57^, ^98^ K_1−_ *_x_*Na*_x_*NbO_3_,[[qv: 97a]] LiNbO_3_,^99^ BaTiO_3_,^100^ BaTi_2_O_5_,^58^ PbTiO_3_,^101^ BaMnO_3_.^59^
Electrospinning methods	PVDF,^63^ PVDF‐TrFE,^66^, ^67^ BaTiO_3_,^68^, ^69^, ^102^ PbTiO_3_,^103^ PZT,^104^ Ba_0.6_Sr_0.4_TiO_3_,^70^ BiFeO_3_,^71^, ^105^ Bi_3.15_Nd_0.85_Ti_3_O_12_,^72^ Bi_3.25_La_0.75_Ti_3_O_12_.^73^
Vapor phase deposition	SbSI^78^
Sonochemical methods	SbSI,^79^, ^80^ Ba_1−_ *_x_*Sr*_x_*TiO_3_,^106^ BiFeO_3_.^107^

## 4 Properties and Applications

Ferroelectric materials exhibit ferroelectric properties as well as piezoelectric and pyroelectric properties, the three interdependent properties of ferroelectric material have been widely exploited for use in a number of functional applications,^10^, ^108^ such as in dielectric capacitors, field effect transistors (FeFET), piezo‐sensors, nonvolatile memory (FeRAM) devices, piezo‐actuators, thermal imaging devices, energy harvesting devices, and electro‐optic devices. Based on the special crystal structures of ferroelectric materials, 1D ferroelectric nanostructures present the typical physical properties of the corresponding bulk materials. With the advent of scanning probe microscopy (SPM) and piezo‐response microscopy (PFM) techniques, our understanding of the mechanisms underlying nanoscale piezoelectricity and ferroelectricity has progressed considerably. Here, we introduce the piezoelectric, ferroelectric, nonlinear optical, and photocatalytic properties related to 1D ferroelectric materials.

### 4.1 Piezoelectric Properties and Applications

Although the understanding of the effect of nanoscale size on ferroelectricity and piezoelectricity is still being formulated, piezoelectric nanostructure applications related to energy harvesting have expanded rapidly since the discovery of piezoelectricity over a century ago.^109^ Piezoelectricity is closely related to the size and dimension of the materials. Piezoelectric coefficients d_33_ is one of the most commonly used parameters in characterization of piezoelectric properties. It represents the displacement due to electric field in specific direction, while the first number of the subscript refers to the electric field direction and the second refers to the direction of stress or strain. **Table** **2** compares the piezoelectric coefficients d_33_ of certain representative ferroelectric materials.

**Table 2:** Piezoelectric coefficients d_33_ of certain representative ferroelectric materials

	d_33_ [pm V^−1^]			
Material	Morphology 1D		2D/film	Bulk
LiNbO_3_			3.2^110^	34.5^111^
KNbO_3_	Nanowire	10.8–27^112^		98^113^
	Nanorods	55^114^		
PbTiO_3_	Nanotube arrays	120^115^		19.3^116^
Pb(Zr_0.6_Ti_0.4_)O_3_			100_(100) orientation_	PZT‐4: 289
			63_(111) orientation_	PZT‐5A: 374
			7_random orientation_ 117	PZT‐6B: 71
				PZT‐7A:150
Pb(Zr_0.2_Ti_0.8_)O_3_			50^118^	PZT‐8:225^119^
b(Zr_0.3_Ti_0.7_)O_3_	Nanofiber	83^120^		
BaTiO_3_	Nanowire	16.5 (d_15_)^121^	30_(100) orientation_ 122	85.6^123^
BiFeO_3_			70_(001) orientation_ 123	
Bi_3.15_Nd_0.85_Ti_3_O_12_	Nanofiber	89[[qv: 68a]]		
Bi_3.25_La_0.75_Ti_3_O_12_	Nanofiber	61^125^	19	
(Na_0.82_K_0.18_)_0.5_Bi_0.5_TiO_3_	Nanofiber	96^126^		
Ba(Ti_0.80_Zr_0.20_)O_3_‐	Nanofiber	180^127^	140	
0.5(Ba_0.70_Ca_0.30_)TiO_3_				
PVDF (β phase)	Nanotube arrays	19.2^128^	14.5	20^129^
PVDF‐TrFE (ratio 56:44)	Nanograss	210^130^	20(LB film)	51
(ratio70:30)			40(spun film)^131^	40
(ratio80:20)				24^132^
SbSI	Nanorod	12^133^		4000^134^
Sb_2_S_3_	Nanowire	2.0 (*d* = 100–200 nm)		
		0.8 (*d* = 50 nm)^135^		

Although is not a ferroelectric in traditional sense, ZnO is the origin of all the piezoelectric relative nanomaterials and the first material for nanogenerator application.^136^ Ferroelectric material is an excellent piezoelectric material, and the piezoelectric coefficients of ferroelectrics are always one order of magnitude larger than those of single piezoelectric materials. With decreases in ferroelectric size, the large surface‐to‐volume ratios of ferroelectric nanostructures naturally result in strong surface effects. Indeed, the tendency to minimize surface energy levels introduces a considerable amount of intrinsic surface stress, i.e., surface tension, in samples of nanodimension, and is one of the most important surface effects considered.^137^ Chen et al.^138^ introduced the time‐dependent Ginzburg–Landau theory to examine the effects of surface tension and axial stress on the piezoelectric behaviors of ferroelectric NWs with radial polarization, and their results clearly show that both the coercive field and remnant strain decrease with increases in surface tension. Axis compressive stress enhances the coercive field and remnant strain, whereas axis tensile stress has the opposite effect. Recently, many well‐known ferroelectric materials have been investigated for energy‐harvesting nanogenerators (NGs) including PZT and BaTiO_3_, with the potential for higher power out puts due to their higher piezoelectric coefficients.^139^

Jung et al.^114^ successfully synthesized high‐quality KNbO_3_ NRs via hydrothermal methods, and the piezoelectricity of these KNbO_3_ NRs was examined via PFM. The measured piezoelectric coefficient (d_33_) of the KNbO_3_ NR was ≈55 pm V^−1^. Subsequently, a piezoelectric KNbO_3_‐based NG was fabricated by spin‐coating a blended KNbO_3_–PDMS (polydimethylsiloxane) composite (volume ratio of 1:100) on a poly(methylmethacrylate) (PMMA) and Au/Cr‐coated Kapton film. The obtained NG produced an open‐circuit voltage of 3.2 V and a closed‐circuit current of 67.5 nA (current density of 9.3 nA cm^−2^) at a 0.38% strain and a 15.2% s^−1^ strain rate. Moreover, the flexible KNbO_3_–PDMS‐based NG presented a nearly frequency‐independent dielectric constant (≈3.2) and low dielectric loss (<0.006) for the 10^2^–10^5^ Hz frequency range. By vertically aligning monoclinic KNbO_3_ NWs on SrTiO3 substrates, Kim et al.^31^ fabricated energy‐harvesting NGs of superior performance relative to orthorhombic NWs. The power densities of the monoclinic‐ and orthorhombic‐NW‐based NGs were reported at 178.0 and 36.3 nW cm^−2^, respectively. Moreover, the monoclinic NWs showed higher voltage and current generation levels.

**Figure 8:**
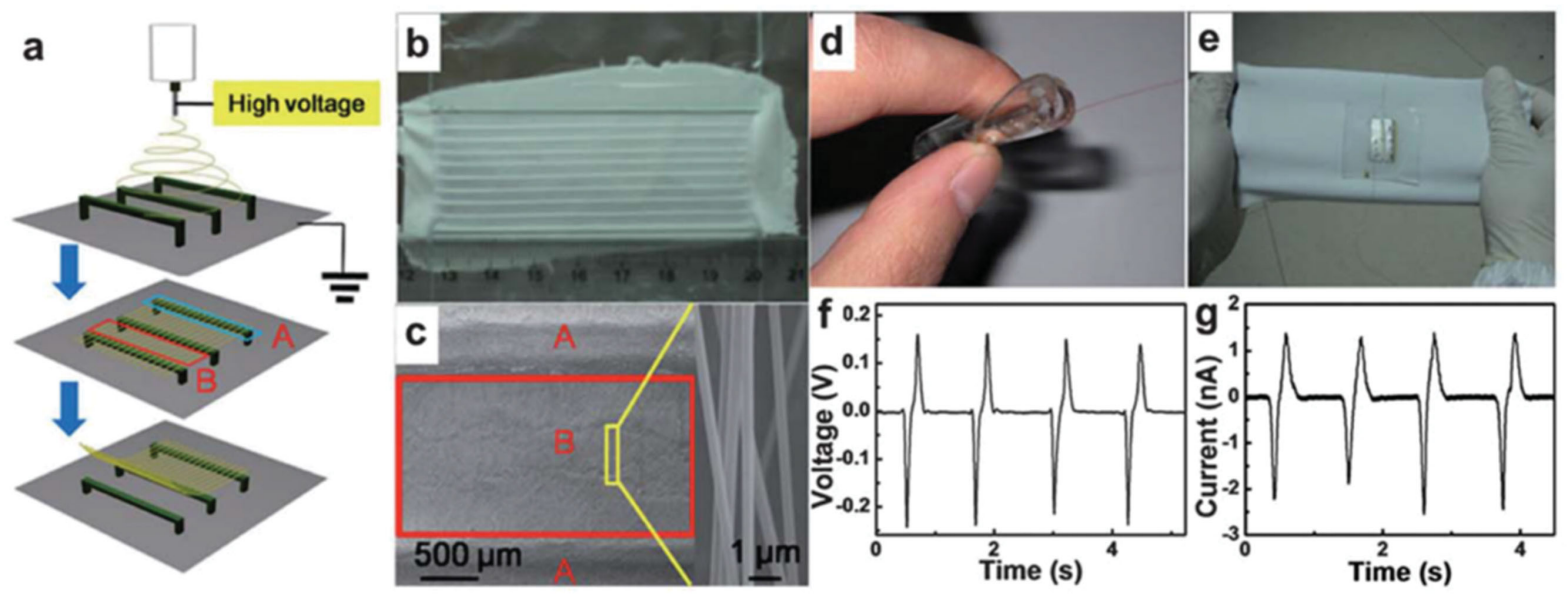
a) Schematic diagram of the electrospinning process of fabricating a PZT textile. Photograph of b,c) SEM images of the PZT textile, respectively. d) Photograph of a tube bent with a substrate‐free PZT textile NG, thus showing its flexibility level. e) Working mode of a wearable NG; the stretch and release of chemical fabric can drive the NG f) output open‐circuit voltage and g) short‐circuit current of a wearable NG, respectively. Reproduced with permission.[[qv: 22a]] Copyright 2012, American Chemical Society.

Ultralong and flexible single‐crystalline PZT‐NWs are among the most widely used piezoelectric material and have been synthesized via hydrothermal methods.^140^ Because of the self‐polarization effects of PZT NWs, these NWs can be used to directly fabricate NGs without further polarization. Nguyen et al.^141^ reported on the fabrication of PZT NW arrays through a combined photolithographic and electrochemical etching method and generated PZT NWs with effective piezoelectric coefficients as high as 145 pm V^−1^. Subsequently, Xu et al.[[qv: 88a]] presented the first report on the chemical epitaxial growth of PZT NW arrays as well as their applications as high‐output energy converters. The NGs fabricated from a single array of PZT NWs produced a peak output voltage level of ≈0.7 V, a current density level of 4 μA cm^−2^ and an average power density level of 2.8 mW cm^−3^. A follow‐up experiment demonstrated that the harvested energy can power a commercial LED. Furthermore, Qi et al.[[qv: 22d]] fabricated stretchable and wearable NGs using wavy PZT ribbons integrated on silicone rubber. The PZT‐based NGs produced a current density of 2.5 μA cm^−2^, which is much larger than that of KNbO_3_‐based NGs. Additional experimental results have shown that the wave amplitudes accommodate order‐of‐magnitude increases in the maximum tensile strain without fracturing. Furthermore, d_33_ values in the buckled regions after polarization have been recorded at ≈180 pm V^−1^, thus constituting a nearly 70% increases from the response in flat regions (75 pm V^−1^).[[qv: 22e]] Therefore, buckled PZT ribbons may serve as wearable or even implantable energy harvesting devices (via encapsulated PDMS).^142^ Using more sophisticated of NGs, Wu et al.[[qv: 22a]] fabricated flexible and wearable NGs using PZT NWs embedded in a polymer (PVP) and textile matrix (chemical fabric), respectively (**Figure** **8**). These NGs can generate 6 V of output voltage and 45 nA of output current, thus providing sufficient power to operate a liquid crystal display and UV sensor. However, challenge remains severe for applications of 1D ferroelectric materials in flexible electric systems, thin‐film materials are still dominate in this field. Recently, a number of related researches have been carried on 1D materials.^143^

In 2014, Koka et al.^144^ successfully produced BaTiO_3_ NRs using barium hydroxide via a hydrothermal process to convert hydrothermally‐grown TiO_2_ NRs grown on conductive fluorine‐doped tin oxide (FTO) substrates. The typical lengths and diameters of the BaTiO_3_ NRs were 1 μm and 90 nm, respectively. The NGs fabricated using the BaTiO3 NRs were accelerated using a shaker table at 1 g (9.8 m s^−2^), generating a peak‐to‐peak open‐circuit voltage of 623 mV, and short‐circuit current density of 9 nA cm^−2^ with a power density of 6.27 mW cm^−3^ on a 120 MΩ load. Similar BaTiO_3_ NW‐based NGs were also demonstrated, but where much longer (40 mm) NWs were used, facilitated by converting extremely high aspect ratio sodium titanate NWs.^145^ These ultralong NWs were shown to produce an output of 390 mV and 1.86 nA, with 192 nW cm^−3^ on a load of 100 MΩ using an acceleration of only 0.25 g.

Li et al.^128^ fabricated a PVDF homopolymer NT array with the desired ferroelectric β‐phase using an AAM template. The effective piezoelectric coefficient d_33_ of the β‐phase PVDF NT array was recorded at 19.2 pm V^−1^, which was significantly larger than the thin‐film counterpart on a solid substrate. Therefore, β‐phase PVDF NTs may be used in piezoelectric sensors and transducers. Furthermore, high piezoelectricity levels have been observed in [(Bi_0.5_Na_0.5_)_0.92_Ba_0.08_TiO_3_] NTs, [Ba(Ti_0.80_Zr_0.20_)O_3_‐0.5(Ba_0.70_Ca_0.30_)TiO_3_] nanofibers,^127^ and BiFeO_3_ NT arrays,^40^ thus indicating their potential use in fabrication piezoelectric sensors and actuators.

### 4.2 Ferroelectric Properties and Applications

Ferroelectric random‐access memories (FeRAMs), have low power usage requirements, fast writing performance levels, high density levels, complete nonvolatility features, and extreme radiation hardness levels,^146^ therefore, they have attracted considerable attention. Based on the reversible polarization of ferroelectrics, various ferroelectric nanostructures have been used in FeRAMs. In addition, ferroelectric polarization can be directly coupled with the channel of a field effect transistor (FET) to form ferroelectric FeFETs, wherein the direction of polarization determines the on‐off state of the device.^147^

**Figure 9:**
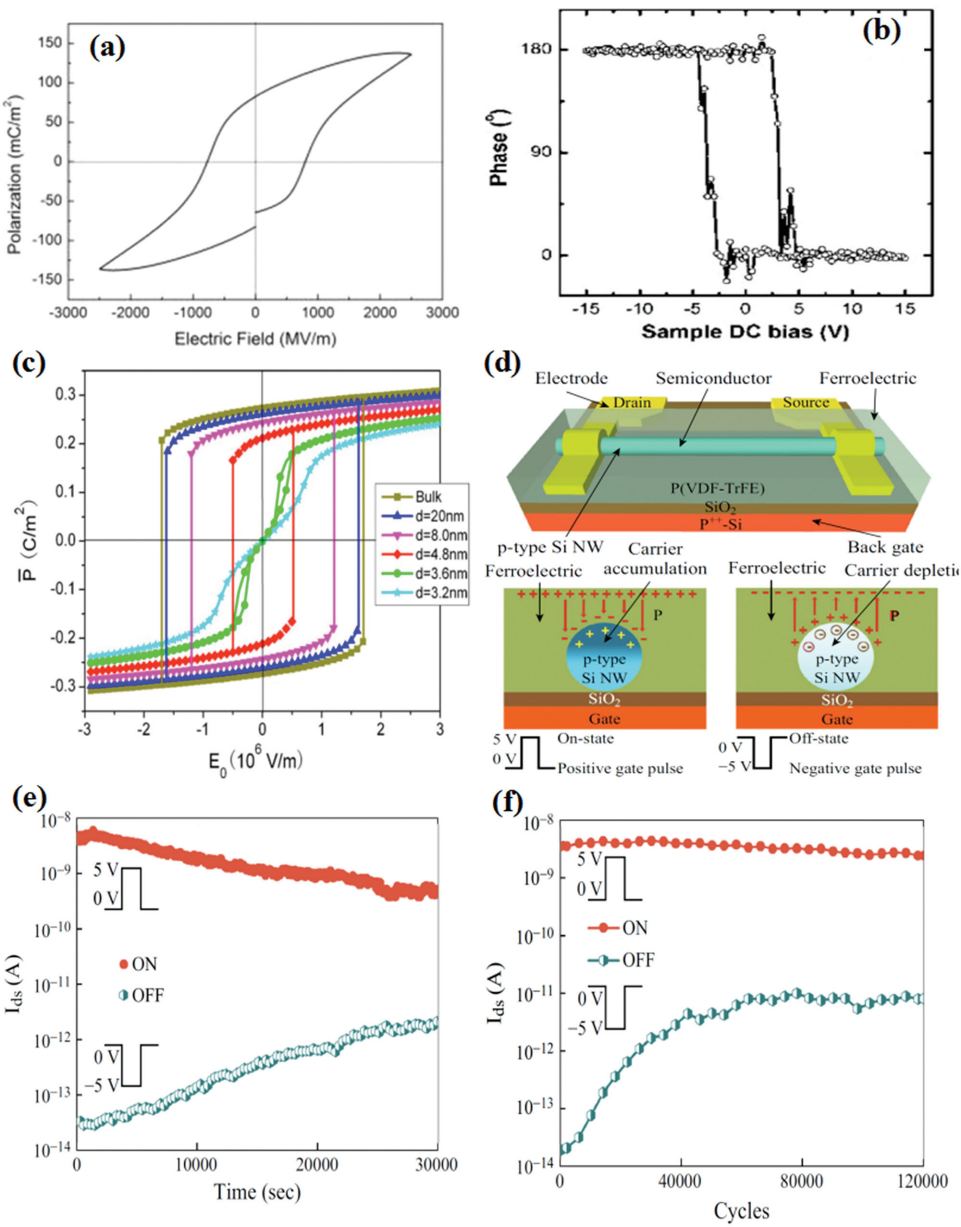
a) P−E hysteresis loop of the released PVDF NT array. b) Lateral PFM in‐field hysteresis phase loop of a BaTiO_3_ NW lying on Au/Pd coated substrate surface; c) Size effects of hysteresis loops of BaTiO_3_ NWs (*T* = 293 K); d) Schematic view and operating mechanism of a back‐gate FeFET‐based nonvolatile memory device; the memory properties of an Si NW‐coated PVDF‐TrFE FeFET‐based device, including the e) retention times and f) endurance tests of the drain currents of drain currents of the Si NW FeFET device. The gate pulse was recorded at ±5 V with a 100 ms pulse width and *V*_ds_ = 0.1 V. a) Reproduced with permission.^63^ Copyright 2011, Royal Society of Chemistry. b) Reproduced with permission.^149^ Copyright 2006, American Institute of Physics. c) Reproduced with permission.^152^ Copyright 2014, American Chemical Society d,e,f) Reproduced with permission.^153^ Copyright 2014, Springer.

PVDF nanofibers^63^ and NT arrays^128^ with the desired ferroelectric β‐crystalline phase have been successfully fabricated, which have dipoles naturally aligned within the crystals and can be reversed through the application of an external electric field. Thus, ferroelectricity in PVDF is caused by its microstructure and β‐crystal formation. **Figure** **9**a shows the polarization‐electric field (P‐E) hysteresis loop of the PVDF NT array. The NT sample showed a high remnant polarization Pr of 82.4 μC m^−2^, which was comparable to the β‐phase PVDF films. However, the coercive field *E*_c_ of the PVDF NTs was significantly higher (796 kV mm^−1^) compared with 1 μm thick PVDF films (115 kV mm^−1^), indicating that the dipoles in the PVDF NTs were much more difficult to switch.^148^

BiFeO_3_ (BFO) is a multiferroic material, and the remnant polarization (Pr) of BFO thin films has been reported in a range from 55 μC cm^−2^ to 80 μC cm^−2^, thus rendering it a promising candidate for FeRAM. Li et al.[[qv: 35a]] reported on the ferroelectric properties of single‐crystalline BFO NWs. The synthesized BFO NWs exhibited spontaneous polarization along the axial and radial directions, which indicates that such ferroelectric BFO NWs have the potential for use in nonvolatile memory application. However, BFO is less preferential because of its low degree of electrical resistivity at RT. This issue can be addressed by replacing the Bi atoms with La and Nd atoms or by replacing the Fe atoms with Mn, Nb, Ti, and Cr atoms.^150^ Guo et al.^45^ reported on the successful preparation of Bi_0.85_Nd_0.15_FeO_3_ (BNF) NT arrays, and the remnant polarization (2Pr) and coercive electric field (2Ec) of the BNF NT arrays at a frequency of 1000 Hz were recorded at 68.7 μC cm^−2^ and 14.2 kV mm^−1^, respectively.

In addition, Tang et al.^73^ reported on the ferroelectricity of bismuth titanates with La‐substituted (Bi_3_ *_._*_25_La_0.75_Ti_3_O_12_, BLT) nanofibers using PFM measurements. The effective piezoelectric coefficient (d_33_) of the BLT fibers was three times larger than the thin‐film counterpart, thus illustrating the advantages of these 1D ferroelectric materials.

BaTiO_3_ is one of the most intensively examined classes of ferroelectric materials. Yun et al.^151^ performed scanned‐probe investigations of the ferroelectric properties of single‐crystalline BaTiO_3_ NWs and found that nonvolatile electric polarization can be reproducibly induced and manipulated on these NWs. The coercive field of polarization reversal was 0.7 kV mm^−1^, and the retention time for induced polarization exceeded 5 d. Therefore, these BaTiO_3_ NWs may be well suited for use in nanoscale nonvolatile memory devices. Figure 9b shows the phase–voltage of a BaTiO_3_ NW, the domain phase exhibits an exactly 180° domain switching feature under the reversal of the external electric field from +15 V to −15 V. In addition, Yun et al. reported on the presence of size‐dependent ferroelectricity in BaTiO_3_ NWs. Hong et al.^152^ applied the Landau–Ginsburg–Devonshire theory to examine the size‐dependent ferroelectric properties of BaTiO_3_ NWs and they showed that the size effects are obvious only when the diameter is less than 20 nm. The ferroelectric properties of BaTiO_3_ NWs, including their Curie temperatures, mean polarization levels, hysteresis loop areas, coercive electric fields, and remnant polarization levels, disappeared when the diameter fell below 3.6 nm (293 K) or 1.2 nm (0 K), and decreased as the NW diameter decreased (Figure 9c). Besides, Tang et al.[[qv: 28d]] reported on the ferroelectricity of bismuth titanates with La‐substituted (Bi_3_ *_._*_25_La_0.75_Ti_3_O_12_, BLT) nanofibers through PFM measurements. The effective piezoelectric coefficient (d_33_) of BLT fibers is three times larger than that of thin‐film counterpart, thus revealing the advantages of 1D ferroelectric materials.

A new nonvolatile memory device based on FeFETs was recently fabricated using a p‐type Si NW coated with omega‐shaped gate organic ferroelectric PVDF‐TrFE.^153^ Excitingly, this memory device exhibited excellent memory characteristics, a low programming voltage level of ±5 V, a high channel conductance modulation between the ON and OFF states exceeding 10^5^, long retention times exceeding 3 × 10^4^ s, and high endurance over 10^5^ programming cycles while maintaining an *I*_ON_/*I*_OFF_ ratio higher than 10^2^ (Figure 9d–f).

### 4.3 Nonlinear Optical Properties and Applications

Light coupling to nanoscale ferroelectrics allows for novel methods of engineering and manipulating coupled degrees of freedom and inducing new avenues of functionality, including photoferroelectric effects and other opto‐mechanical responses.^154^ 1D NWs with diameters comparable to the optical wavelength, exhibit modified light‐matter interactions such as field enhancements, light‐confinement features, waveguiding patterns, and enhanced nonlinear optical responses.^155^

**Figure 10:**
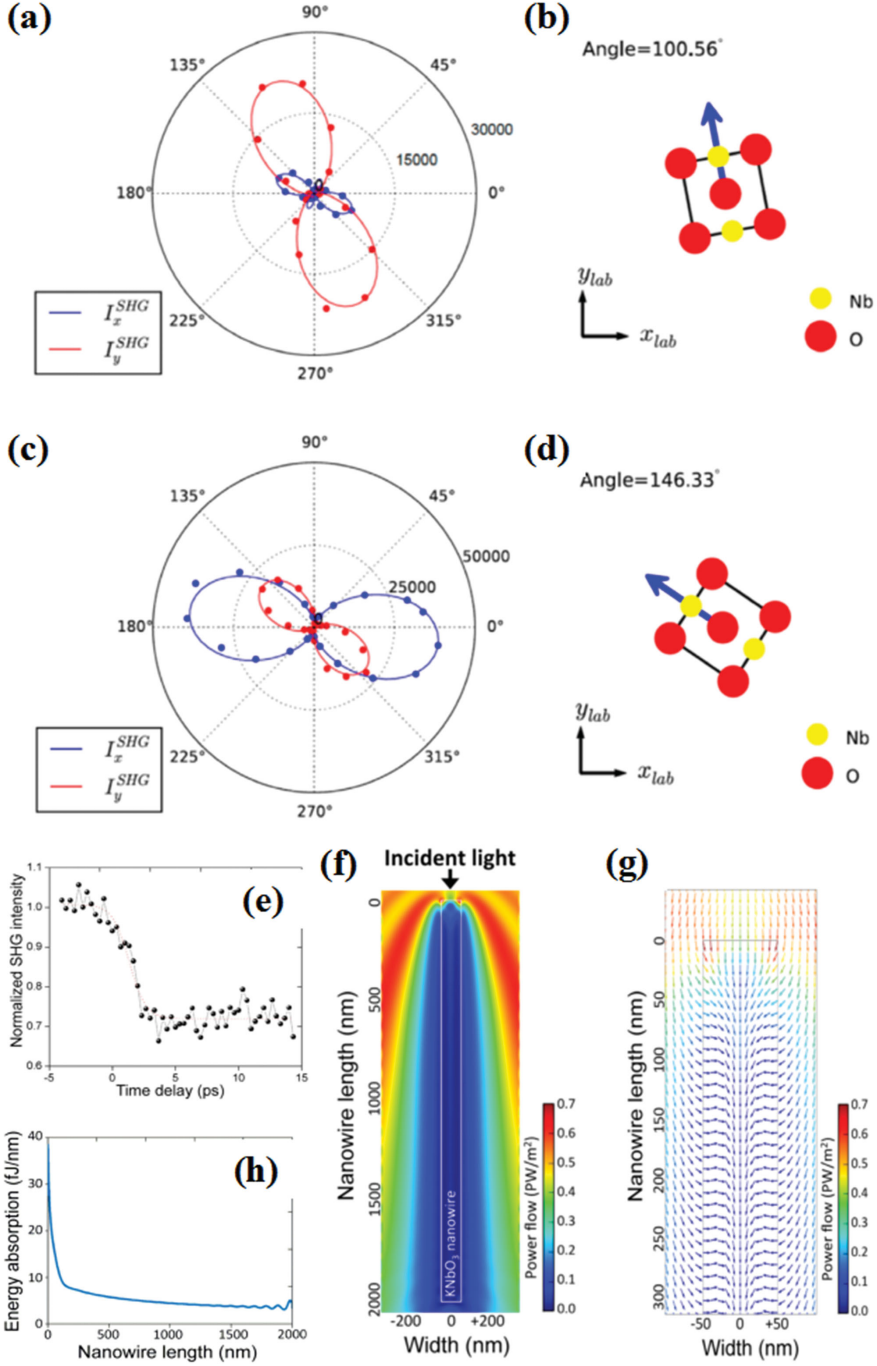
a) Polarization dependence of SHGs observed from a trapped NW with a polarizer parallel to the x‐axis (blue) and yaxis (red). b) Schematic unit cell of the NW used at an azimuthal angle of 100.56° for the spontaneous polarization direction (blue arrow). c) Polar plots from another trapped NW. d) Schematic unit cell of the NW used in (c) at an azimuthal angle of 146.33° (blue arrow). e) Time‐resolved SHG signals of a trapped NW as a function of pump–probe time delays upon photoexcitation (guideline in red dots). f) 3D finite difference time domain (FDTD) simulation showing the amplitude of the power flow over a free‐standing NW(white rectangle; 100 nm in diameter and 2 μm in length) in water. Incident plane wave (*E* = 0.53 GV m^−1^, 50 fs) propagating along the long axis of the NW. g) Calculated directions of the power flow shown in (f) and h) energy absorption distribution per pulse within successive 1 nm slices as a function of the NW length. Reproduced with permission.^156^ Copyright 2014, American Chemical Society.

1D KNbO_3_ NWs, especially those of the low‐symmetry monoclinic phase, exhibit enhanced nonlinear optical response levels and have thus attracted considerable interest. Kim et al.^31^ examined the nonlinear (NLO) properties of KNbO_3_ NWs through second harmonic generation (SHG). The emission spectrum shows that 1064 nm laser radiation levels peak at 532 nm, thus revealing the frequency‐doubling capabilities of KNbO_3_ NWs. Moreover, Nah et al.^156^ presented femtosecond measurements of the light‐induced polarization dynamics within an optically trapped KNbO_3_ NW. When the NW is optically trapped, the long axis of the NW is oriented along the optical axis, which results in a 45° inclination in spontaneous ferroelectric polarization to the optical axis. Large amplitudes, reversible photoinduced modulations in nonlinear optical properties and polarization are observed in single NWs at megahertz repetition rates associated with enhanced light absorption within NWs, with a subwavelength diameter (**Figure** **10**).

**Figure 11:**
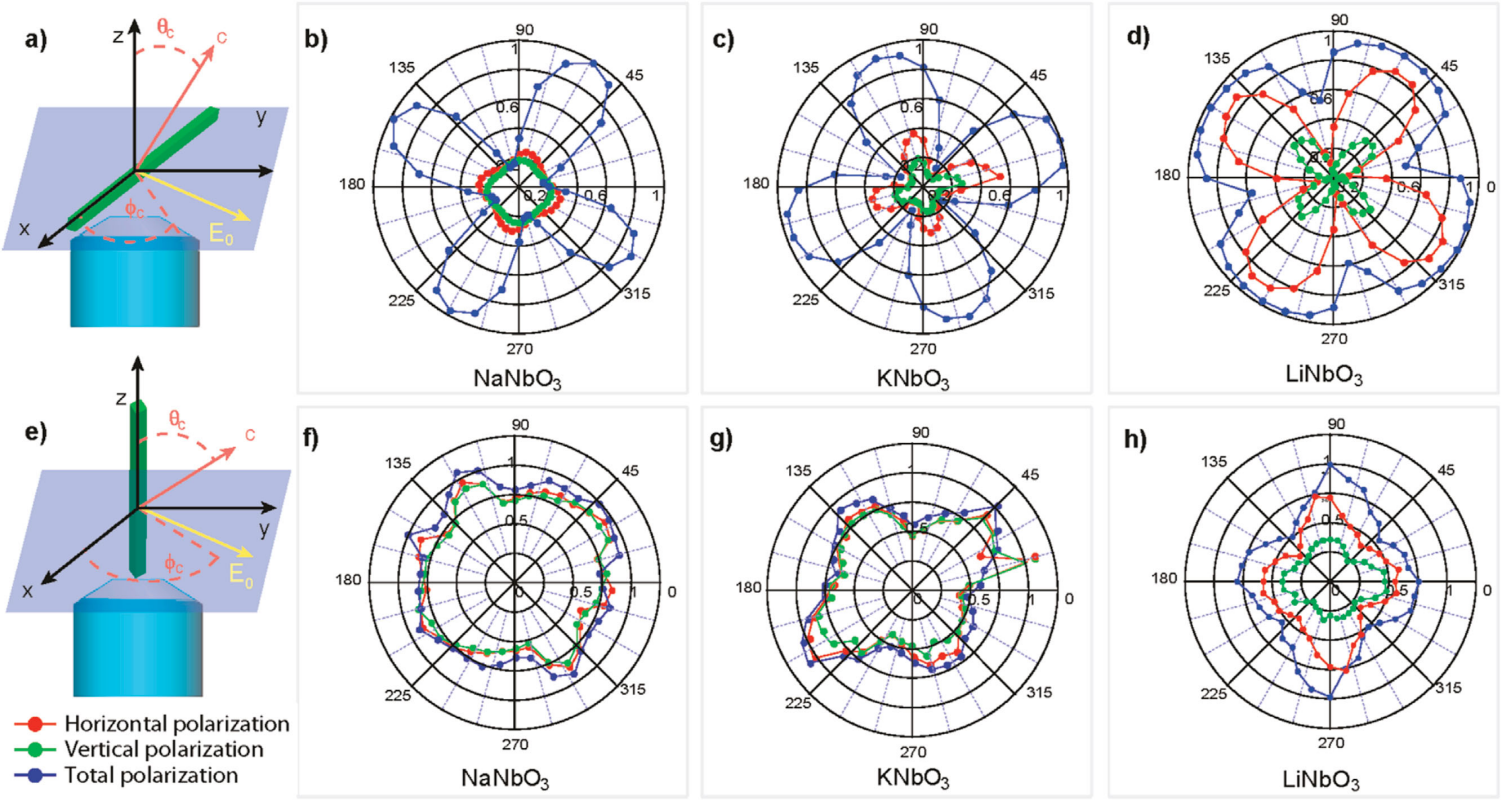
a) Schematics of experimental geometries employed in the polarization experiments on the NWs stuck to glass substrate, where *θ*_c_ denotes an angle of inclination of the crystal c‐axis relative to the NW geometric axis and *φ*_c_ denotes an azimuth of the incidental linearly polarized light. Polar plot of the normalized SHG signal from the NWs stuck to glass substrate for the three experimental conditions: b) NaNbO_3_, c) KNbO_3_, and d) LiNbO_3_. e) Schematics of the experimental geometries employed in the polarization experiments on the optically trapped NWs. Polar plot of the normalized SHG signal from the optically trapped NWs under three experimental conditions: f) NaNbO_3_, g) KNbO_3_, and h) LiNbO_3_. Reproduced with permission.^157^ Copyright 2011, American Chemical Society.

More recently, Dutto et al.^157^ performed systemic studies on the SHG properties in XNbO_3_ (X = Li, Na, K) NWs. All three of NWs exhibited strong nonlinear responses and the LiNbO_3_ NWs exhibited the strongest response. Moreover, the polarization responses of the SHG signals in all three types of XNbO_3_ NWs for the two geometries were studied, and the intensity of the SHG signal was polarization‐dependent and a function of the degree of XNbO_3_ NWs structural order (**Figure** **11**). The nonlinear properties of the above‐listed NWs were examined, and each XNbO_3_ NW was found to be suitable for different applications, with LiNbO_3_ NWs effective imaging markers because they generate the highest nonlinear response and KNbO_3_ NWs effective opto‐mechanical probes because of their high trapping stability levels and relatively high nonlinear responses. Although all three XNbO_3_ NWs present similar in‐coupling wave‐guiding efficiency levels as potential waveguides, theNaNbO_3_ NWs are regarded as the superior choice because they generate the highest aspect ratio.

### 4.4 Photocatalysis Properties and Applications

Over the last decade, 1D nanostructures have received considerable attention with regard to their ability to photocatalytically degraded organic pollutants, and 1D TiO_2_ nanostructures, (e.g., TiO_2_ NBs), have been studied because of their low number of grain boundaries, fast charge transfer dynamics and high specific surface area levels, which can improve photocatalytic performance levels.^158^ It is well known that photocatalytic activity originates from the oxidation of photoinduced holes and reduction of photoinduced electrons. Thus, 1D nanostructures can enhance photocatalytic performance levels by adjusting the directions and paths of photogenerated charge carriers via quantum confinement while minimizing electron–hole recombination.^159^ Moreover, 1D nanostructures cover a large specific surface area relative to their bulk counterparts and generate more active sites that can produce the desired photocatalytic reactions, thus enhancing efficiency levels.

**Figure 12:**
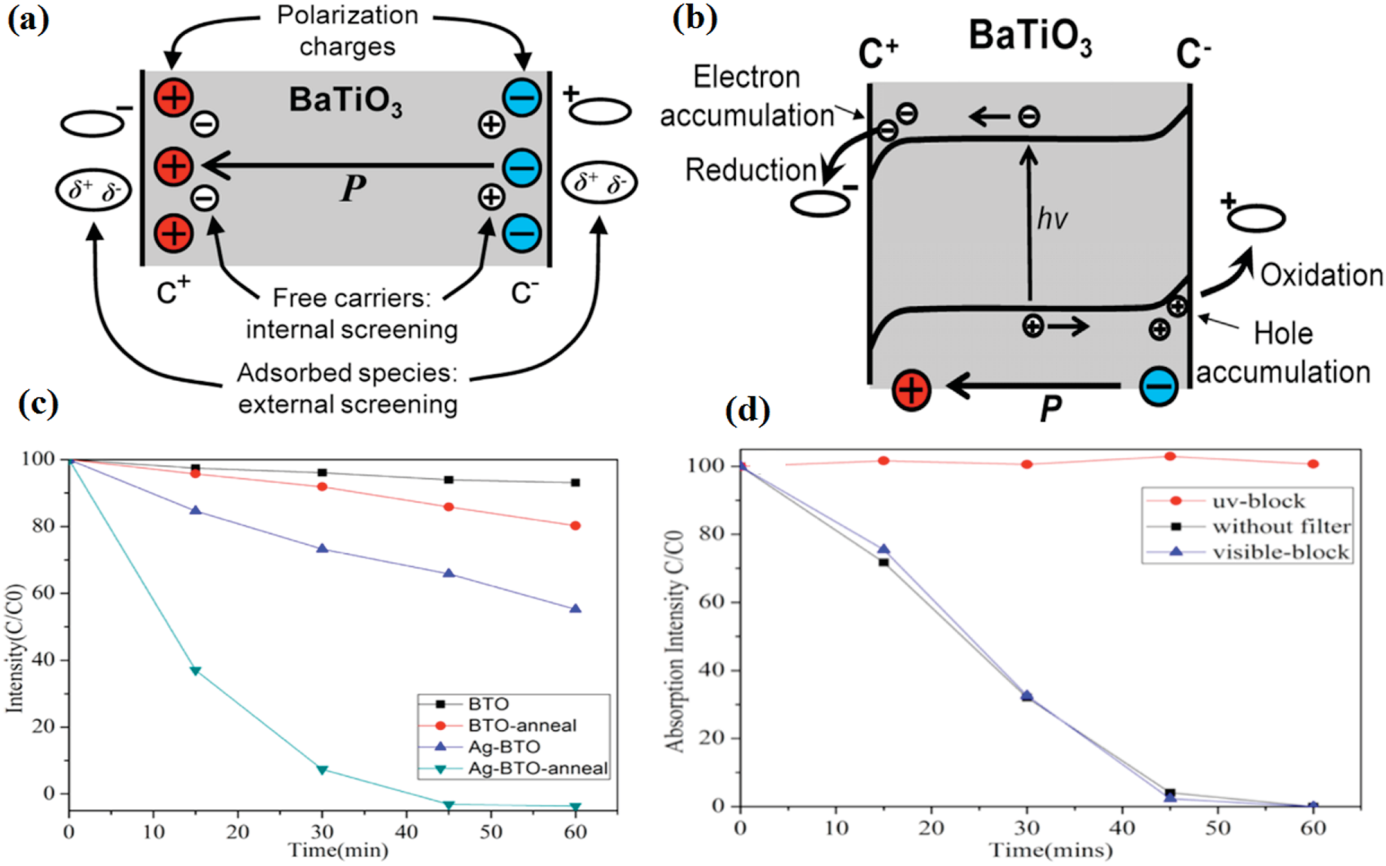
Schematic of a ferroelectric material showing a) the internal polarization and screening mechanisms and b) the effect of free carrier reorganization on the band structure and photoexcited carriers. c) Photodecolorization profiles of RhB (Rhodamine B) with different catalysts under solar simulators and d) photocatalytic decolorization of RhB using UV‐visible‐blocking filters via Ag‐BaTiO_3_‐anneal. Reproduced with permission.^160^ Copyright 2013, American Chemical Society.

Recently, reports have shown that the spontaneous polarization of ferroelectric materials may facilitate the design of high active photocatalysts by promoting the separation of photoexcited carriers. A photocatalytic system composed of Ag‐loaded BaTiO_3_ was introduced by Dunn and co‐workers,^160^ who found that photocatalytic activity levels were enhanced as a result of the effects of ferroelectricity on carrier separation and stern layer formation. The effect of the ferroelectric characteristics on the separation of charge carriers, which is similar to the p−n junction of a typical photovoltaic structure or other diode structure, inhibits the recombination of holes and electrons, thus increasing the lifetime of the photoinduced charge carriers. Moreover, catalyst performance levels can be further enhanced through the development of a nanostructured metallic (Ag) coating on the catalyst surface (**Figure** **12**a–d). These results demonstrated that ferroelectric materials can act as effective photocatalysts. Additional detailed information is provided in the reference section. Although the ferroelectric material (BaTiO_3_) was not a 1D nanostructure, it still provided us with valuable information.

KNbO_3_ is a ferroelectric material that is commonly used as a photocatalyst because it is nontoxic, cost‐effective, and highly stable under light illumination.^161^ Zhang et al.^162^ performed an in‐depth study on the relationships between the structure and photoreactivity of ferroelectric KNbO_3_ NWs and their respective orthorhombic and monoclinic polymorphs. The experimental results clearly showed that *ο*‐KNbO_3_ NWs present RhB (Rhodamine B) degradation rate that are twofold higher than those of *m*‐KNbO_3_ NWs, which can be attributed to the synergy between the delicate atomic structural variation‐derived ferroelectric polarization and electronic structures.

Fu et al.^163^ performed a detailed theoretical study on the structural, electronic, and optical properties of perovskite CaTiO_3_ bulk and NWs using pseudo potential density‐functional theory calculations. The electronic structure calculations showed that the CaTiO_3_ NW band gaps are heavily modified compared with those of the bulk. The optical property calculations indicated that the absorption edge of the NWs shifted toward the red‐light region. These theoretical results suggest that perovskite CaTiO_3_ NWs may be used for visible light photocatalysis applications, such as solar‐assisted water splitting reactions.

### 4.5 Ferroelectric‐Photovoltaic Effect and Applications

Ferroelectric‐photovoltaic (FE‐PV) effect was discovered about half a century ago in a variety of ferroelectric materials without central symmetry in which a steady photovoltaic response (photovoltage and photocurrent) can be generated along the polarization direction.^164^ The FE‐PV effect is distinctly different from the typical photovoltaic (PV) effect in semiconductor p–n junctions in that the polarization electric field is the driving force for the photocurrent in FE‐PV devices, in which a homogeneous ferroelectric material is used as a light absorbing layer.^165^ Thus, there exists two unique and important characteristics of FE‐PV devices, one is that the photocurrent direction can be switched by changing the spontaneous polarization direction of a ferroelectric material with the electric field, the other is the anomalous photovoltaic (APV) effect, i.e., the output photovoltage, which is proportional to the magnitude of electric polarization and electrode spacing,^166^ can be a few orders of magnitude larger than the band gap of the ferroelectric materials.^167^

**Figure 13:**
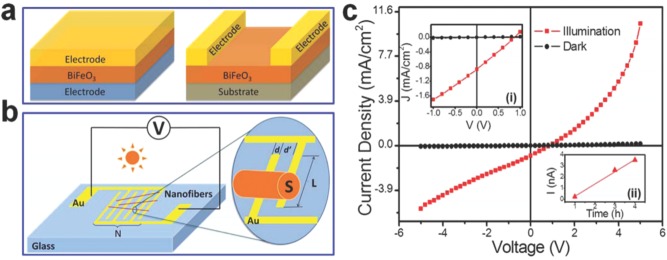
a) Schematic setup for the measurement of thin film‐based photovoltaic devices: left, device with a parallel capacitor configuration; right, device with a laterally aligned interdigital electrodes. b) Schematic setup for the measurement of random BFO nanofiber‐based photovoltaic devices. c) The photoresponse profile of the nanofibers. Inset (i) shows expanded view of current density behavior around zero‐bias. Inset (ii) shows averaged photocurrent after several measurements for different deposition time from 1 to 4 h. Reproduced with permission.^71^ Copyright 2015, American Chemical Society.

To date, FE‐PV effect has been studied in the LiNbO_3_ family,[[qv: 167a]],^168^ BaTiO_3_,[[qv: 167a]] PZT family,^169^ and BiFeO_3_ family.[[qv: 166c]],^170^ However, most studies are focused on thin‐film ferroelectric materials. As predicted, 1D ferroelectric nanostructures, where the unit cell and its corresponding ferroelectric/piezoelectric properties are supposed to be significantly influenced by the surface effect,^171^ might bring considerable advances to the research of FE‐PV devices. In 2015, Fei at al.^71^ successfully synthesized BiFeO_3_ (BFO) nanofibers via a sol–gel based electrospinning process followed by thermal treatment and a FE‐PV device using laterally aligned BFO nanofibers and interdigital electrodes (IDE) was developed. For comparison, two major types of BFO‐thin‐film based FE‐PV devices were discussed, devices with a parallel capacitor type of structure (left in **Figure** **13**a), could offer a relatively large photocurrent but low voltage, whereas devices with an in‐plane electroded capacitor structure (right in Figure 13a) could generate a relatively high voltage but small photocurrent. To solve these problems, the authors developed BFO‐nanofiber based FE‐PV devices with a configuration of nanofibers/IDE/substrate (Figure 13b) and found that the BFO nanofibers exhibits an excellent FE‐PV property with the photocurrent several times larger than the literature data obtained on BFO thin films. The reasons for the nanofibers showing enhanced FE‐PV properties could be related to two factors. First, the nanofibers are free‐standing, whereas the thin films are mechanically clamped by substrates. Second, the nanofibers could trap more photons due to geometric confinement.

## 5 Summary and Prospective Work

Recently, growing number of research papers related to theories, syntheses, properties, and applications of 1D ferroelectric nanostructures have been published, thus suggesting the potential of these structures. Therefore, we have discussed recent developments related to the synthesis of these materials and presented their practical applications. Compared to physical methods, chemical methods are more cost‐effective for the synthesis of 1D ferroelectric nanostructures and present a greater potential for mass production. The most commonly used chemical methods are hydrothermal, sol–gel template‐assisted, molten salt and electrospinning methods. Hydrothermal methods can fabricate highly crystalline stoichiometric nanostructures and generally produces single‐crystalline NWs. Template‐assisted methods use templates with pores/channels and controllable shapes and sizes, and they are regarded as the simplest methods of preparing 1D ferroelectric nanostructures. The molten salt method is distinct in its use of a precursor, which has a significant effect on the product morphologies and dimension. Electrospinning is likely the most versatile method of fabricating polycrystalline NWs and nanofibers. Currently, because the synthesis processes require multiple processing steps, and are sensitive to small changes in synthesis conditions, the experimental parameter optimization is often necessary and complex. It brings the biggest challenge in the use of 1D ferroelectric nanostructures for practical application that is the scaling up production. Therefore, new powder processing‐based techniques are highly desired to realize mass and stable production of 1D ferroelectric nanostructures.

Potential applications of 1D ferroelectric nanostructures are truly impressive and broad, including nanogenerators, nonvolatile memory devices and photocatalytic degradation of organic pollutants. Recently, breakthroughs in the control of ferroelectricity levels in 1D ferroelectric nanostructures have been achieved, thus generating new potential uses for nanoscale ferroelectric devices, particularly for FeFETs. However, such practical applications are far from being realized, and several problems cannot be neglected. First, further studies should be performed on the growth mechanisms so that 1D ferroelectric nanostructures can be prepared with predictable shapes and controllable sizes. The controlled morphology of nanostructures synthesized over large areas under reproducible synthesis conditions is essential for their increased application. Second, in order to reliably integrate 1D ferroelectric nanostructures into other components to realize the specific properties, a thorough understanding of their relevant physical properties is required. However, in such a nanometer scale, it is difficult to finish the traditional physical property measurement of the materials. More methods and equipment are required to collect these physical properties. Third, lead‐free compositions are promoted to alternate the lead‐based ferroelectric nanostructures. However, in general, the lead‐free ferroelectric materials possess much weaker ferroelectric properties compared to those of the lead‐based ones. More attention should be paid to understand the fundamental reason of this different property, and trying to overcome the barrier with lead‐free ferroelectric materials. At last, sufficient tests should be conducted prior to their application to support the long‐term stability of nanostructures. The stability of the nanostructure in the devices should be improved and confirmed.
